# Diagnostic Techniques of Soil-Transmitted Helminths: Impact on Control Measures

**DOI:** 10.3390/tropicalmed5020093

**Published:** 2020-06-05

**Authors:** Mirabeau Mbong Ngwese, Gédéon Prince Manouana, Paul Alvyn Nguema Moure, Michael Ramharter, Meral Esen, Ayola Akim Adégnika

**Affiliations:** 1 Centre de Recherches Médicales de Lambaréné (CERMEL), Lambaréné BP: 242, Gabon; mmbongngwese@tuebingen.mpg.de (M.M.N.); gedeon.manouana@cermel.org (G.P.M.); alvynhyou@gmail.com (P.A.N.M.); 2 Max Planck Institute for Developmental Biology (MPI), Department of Microbiome Science, Max-Planck-Ring 5, 72076 Tübingen, Germany; 3 Institut für Tropenmedizin, Eberhad Karls Universität Tübingen, D-72074 Tübingen, Germany; meral.esen@uni-tuebingen.de; 4 German Centre for Infection Research (DZIF), partner site Hamburg-Luebeck-Borstel, D-20359 Hamburg, Germany; ramharter@bnitm.de; 5 Department of Tropical Medicine, Bernhard Nocht Institute for Tropical Medicine and I. Department of Medicine University Medical Center Hamburg-Eppendorf, D-20359 Hamburg, Germany; 6 German Center for Infection Research (DZIF), D-72074 Tübingen, Germany

**Keywords:** diagnostics, intestinal helminths, soil-transmitted helminths, control measures

## Abstract

Soil-transmitted helminth (STH) infections are common in the tropical and subtropical countries. The burden of disease is highest in endemic areas with limited access to good quality water supply and poor sanitary conditions. Major approaches to control and reduce morbidity caused by worm infections include the periodic deworming of pre-school and school-aged children with anthelminthic drugs. Population-based studies and individual patient management including interventional studies can only be successful when accurate diagnostic techniques are used. The lack of appropriate diagnostic tools providing accurate results concerning both infectious status and intensity of infection—as these two factors vary in regions of low infection intensities—is a major challenge. Currently, available techniques show limited sensitivity and specificity and as such, a combination of several techniques is usually used to diagnose the large variety of parasite species. The objective of this review was to describe the advantages and disadvantages of the different available techniques for the diagnosis of STH infections and to highlight their use in control programs.

## 1. Global Burden of Disease and Importance in Epidemiology

There are four species of soil-transmitted helminths (STHs) that cause infections in humans, namely *Ascaris lumbricoides*, *Trichuris trichiura* and the hookworms (*Necator americanus* and *Ancylostoma duodenale*). They are considered as neglected tropical diseases (NTDs) by the World Health Organization (WHO). Although *Strongyloides stercoralis* is not included in this list of NTDs, its geographical overlap with other STHs and the morbidity related to this parasite also make it an important STH. These parasites are associated with poverty, causing a significant morbidity measured in disability-adjusted life years (DALY’s) lost [1,2]. Global estimates suggest that about 1.5 billion people are infected with STHs worldwide. Two hundred and seventy (270) million are preschool children and over 568 million are school-aged children that require treatment and prevention interventions. People harboring heavy infections have a higher morbidity, while people carrying light intensity infections are usually asymptomatic. Thus, heavily infected people particularly have debilitating outcomes usually resulting in a variety of specific and unspecific adverse effects like reduced physical growth and cognitive impairment in children [3], as well as anemia and intestinal occlusion.

Recent estimates suggest that these four STHs infect over 700, 508 and 480 million people worldwide respectively [4]. The highest prevalences are recorded in tropical countries. The total annual number of deaths due to STHs is estimated to be higher than 135,000. Clusters of infections are more common in crowded households [5]. Three principal conditions contribute to the transmission of STHs: soil contamination by human or animal feces; favorable conditions for the eggs/larvae to survive on the soil, the survival of eggs and skin contact with contaminated soil or oral infestation by consumption of contaminated soil, water and /or food [6].

The most vulnerable groups are mainly children of school age between the ages of 5 and 15 years, as well as pregnant women [7,8,9]. Infections are higher in endemic countries with inadequate sanitary conditions, the absence of portable water and limited healthcare facilities [7,10,11]. The risk of infection is higher in farmers during their routine agricultural work, and women and children during their domestic and recreational activities where they are contact with contaminated water [11]. Strategies aimed at controlling STHs have seen a rise in recent decades, and they principally involve the integration of control programs of multiple tropical diseases [10,12,13,14,15]. Another approach involving large-scale or mass drug administration (MDA) targeting high-risk groups has been widely used to reduce worm burden. The WHO recommends preventive chemotherapy, i.e., single-dose anthelminthic treatment given annually or biannually without a prior diagnosis to young children, preschool and school-aged children living in settings where the baseline prevalence of STHs is >=20% [16]. This strategy has already proven to be useful [17,18]. The success of such MDA could be more accurately monitored through the measurement of infection intensities by the use of very sensitive diagnostic tools. Several methods exist for the laboratory diagnosis of STHs including: Kato-Katz (KK), formol-ether (FE), sodium nitrate flotation (SNF), direct examination (DE), Kogar agar plate (KAP), merthiolate-iodine-formaldehyde (MIF), Baermann, McMaster, Harada-Mori and recently developed flotation, translation and centrifugation (FLOTAC) techniques and molecular diagnostic techniques. Examples of these molecular techniques include the polymerase chain reaction (PCR) and Loop-mediated isothermal amplification (LAMP). Each of these techniques has shown promising outcomes in detecting different parasite species, although some have very low sensitivities in providing accurate results especially in light-infection and poly-infection settings [19].

## 2. Choice of Diagnostic Technique

The evaluation of the efficacy, effectiveness and the disease elimination of interventions as well as control in the community and in endemic areas strongly depends on the accuracy of the diagnostic tools which are defined by their sensitivity and specificity [20,21]. Traditionally established methods that are used to detect parasitic elements have always relied on microscopy. However, the detection of parasites by microscopy in each sample is not always achieved, even when subjects are heavily infected. There are several factors that account for this difficulty including but not limited to: possible methodological problems, eggs are not evenly distributed throughout the feces, egg numbers may be too low for detection in stool, amount of stool sample could affect the number of eggs present in the sample, the cyst or ova excreted intermittently or the samples are not transported or stored properly [22,23]. In highly endemic areas, the focus is on the prevention of morbidity and therefore the use of less sensitive techniques is usually sufficient. On the other hand, when the goal is to evaluate the prevalence for surveillance and elimination, the use of highly sensitive methods is required. However, these techniques are expensive and pose an obstacle in resource-limited settings. This has forced many control strategies in these regions to focus on MDA and the use of cheaper techniques with limited sensitivities to diagnose and treat infections. More so, since clinical symptoms are too unspecific for the diagnosis of helminths infections, diverse diagnostic approaches ranging from serology (detection of antibodies and antigens), microscopy (detection of eggs/cysts/larvae/oocysts) or radiology and molecular techniques can be employed.

Mass drug administration (MDA) is a well investigated interventional tool to control several parasitic diseases in endemic areas. Although MDA is usually done without diagnostic tools, their effectiveness can be reliably measured if used in conjunction with a sensitive diagnostic methodology. On the other hand, test and treat strategies require the use of diagnostic methods. Unfortunately, the available methods pose variable levels of limitations concerning sensitivity, specificity, cost-effectiveness, personnel skills, and infrastructure. This is problematic in poor countries with limited resources (human and financial), thus most test and treat control programs carried out in such areas are forced to choose a cheaper and cost-effective diagnostic tool with potentially limited sensitivity and specificity. The consequences are evident, despite the initiation of such antiparasitic interventions in many endemic areas, so the burden of infection remains high. To meet the millennium development goals, such interventional programs should be implemented at a large scale and the measurement, evaluation and alignment of their success will rely on the use of the most effective (sensitive) diagnostic tools.

**Figure 1:**
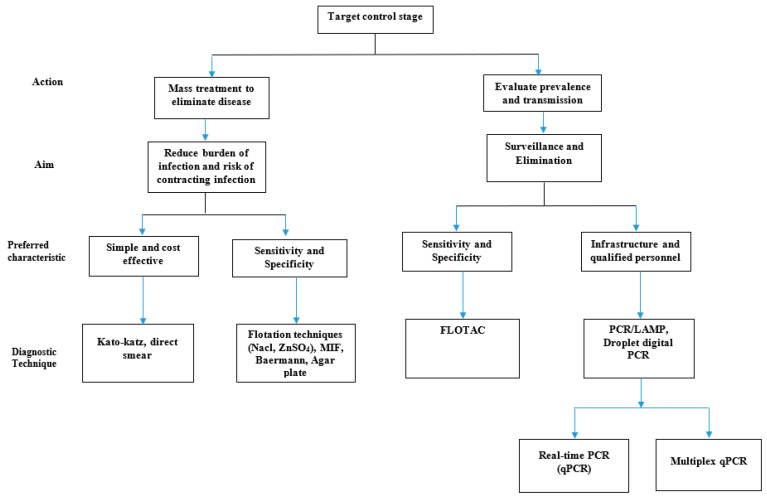
Suggested use of diagnostic techniques for helminth control.

This review aimed to describe the currently available laboratory microscopic and molecular techniques, their advantages, disadvantages, and possible improvements. In Figure 1 we present a flow chart to guide the choice of a diagnostic technique.

## 3. Methodology

For this review, we performed a web-based search for original articles and reviews published using PubMed and Google scholar web. We also obtained information from textbooks. Our search included articles published between 2000 and 2019. However, we also included articles as far back as the 1930s which contained original information when the different techniques were developed. Keywords included diagnostics, intestinal helminths, soil-transmitted helminths, control measures and technique names (e.g., Kato-Katz, FLOTAC, Baermann, Mc Master, Harada-Mori, Coproculture, formol-ether, Kogar agar plate, sodium nitrate flotation, merthiolate-iodine-formaldehyde, and PCR).

## 4. Diagnostic Techniques

### 4.1. Direct Examination

**Figure 2:**
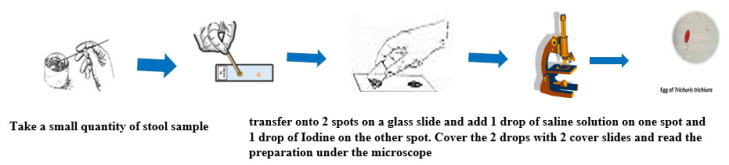
Operating steps for the direct smear technique. Adapted from (WHO 1994).

The direct microscopic examination of feces is essential to detect parasitic elements such as the larvae of *Strongyloides stercoralis* which are motile. It is also usually adequate to detect high concentrations of the eggs of helminth infections with *Ascaris lumbricoides*. The main advantage of this method is that it is rapid and inexpensive. However, it is only semi-quantitative, and it is not often used in control programs. It is more widely used in the routine analysis to detect protozoan parasites such as the trophozoites of *Entamoeba histolytica, Giardia lamblia* and more rarely *Balantidium coli.* It involves emulsifying a small quantity of fresh stool in one drop of saline on a microscope glass slide. A thin smear preparation is obtained by placing a cover glass on the emulsified stool and examined under a light microscope to detect the eggs/larvae/trophozoites of parasite species. An eosin or iodine preparation is also necessary to identify the cysts/oocysts of intestinal protozoa [24]. Figure 2 shows the operating steps of the direct examination [25].

### 4.2. Kato-Katz Technique

**Figure 3:**
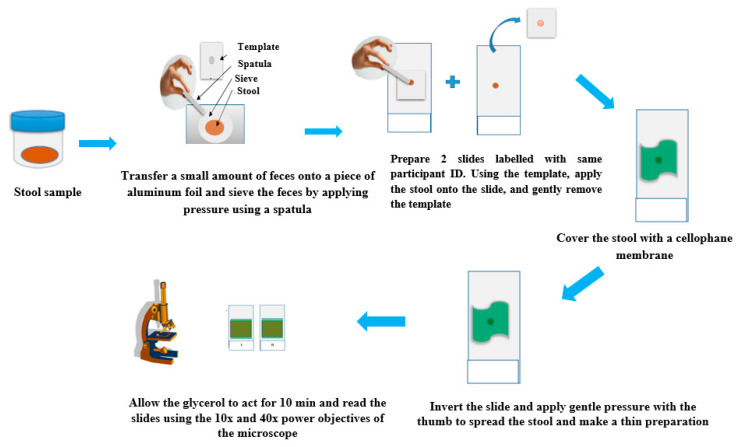
Operating steps for the Kato-Katz technique. Adapted from open source web images.

The Kato-Katz technique is the WHO “gold standard” that is widely used to assess the prevalence and infection intensity of STHs. Amongst the copro-microscopic methods, Kato-Katz has several advantages including; high sensitivity, egg quantification, cost effectiveness and requires minimal infrastructure [26]. It is possible to stratify infection intensities using egg counts and cut-off values [11]. For the Kato-Katz technique, the sieved feces sample (approximately 41.7 mg, 20 mg, or 50 mg depending on the size of the template) is placed on a glass slide. The preparation is covered with a piece of cellophane soaked in glycerol. Subsequently, the slide is inverted and gently pressed down resulting in a thin smear. The added glycerol serves to ‘clear’ the fecal material (fat) from around the eggs. Hookworm eggs require about 30 min for this step, while for the other species, the reading of the slide under the microscope can be done after 1 to 24 h The eggs are then counted under the microscope and the count expressed in per gram of feces [11,26]. Figure 3 shows the operating steps of the Kato-Katz technique.

### 4.3. Formol-Ether Concentration Technique

**Figure 4:**
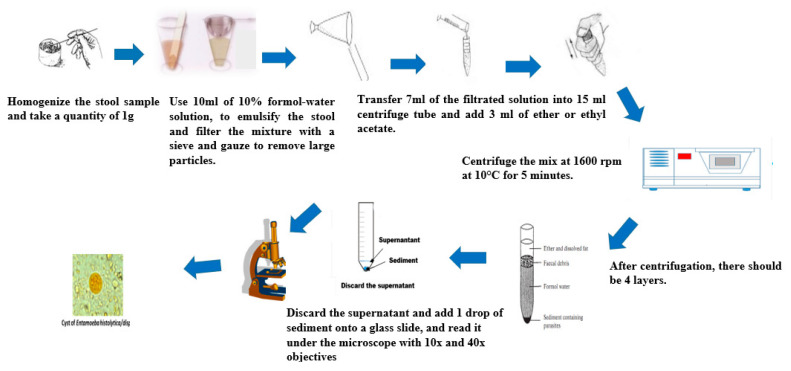
Formol-ether sedimentation of parasites after centrifugation. Adapted from Cheesbrough 2009, WHO 1994.

The formol-ether-concentration method is commonly used in specialized laboratories [27] for the diagnosis of STHs. The main advantage is that it is fast, and it allows for the concentration of a range of fecal parasites. Both fresh and preserved feces can be used with this technique. The use of formol inactivates the organisms and thus minimizes the risk of laboratory-acquired infection from fecal pathogens [28]. STHs as well as intestinal protozoa can be diagnosed with this technique. When used in combination with the Kato-Katz method, the diagnostic sensitivity for helminths is greatly improved [29]. The stool samples can be fixed with either sodium acetate-acetic acid-formalin (SAF) [27], or diluted formalin [30], to allow for sample storage and retrospective analyses. An alternative technique using acetone has been described [31]. Several modifications of the technique have been made over the years. The Ridley modified method [32] involves emulsifying the feces in formol water, followed by straining the suspension to remove large fecal particles. After adding ether or ethyl acetate, the mixed suspension is then centrifuged. The parasitic elements, cysts, oocysts, eggs, or larvae are fixed and sedimented, while the fecal debris are suspended in the layer between the ether and the formol water. The entire sediment is further examined under a light microscope to detect and count the parasite. Figure 4 shows the operating steps of the formol-ether-concentration method.

### 4.4. Agar Plate Culture Technique

The excretion of the larvae of *S. stercoralis* is usually scant in light-intensity infections. This makes the detection of these larvae difficult. [33]. Two techniques have been described as suitable for the diagnosis of *S. stercoralis* and hookworm infections, namely the agar plate culture technique [34] and the Baermann technique [35]. This method requires an agar media (prepared with 1.5% agar, 0.5% beef extract, 1.0% peptone and 0.5% Nacl). Ten milliliters (10 mL) of the prepared medium is transferred into a 150 mm × 15 mm Petri dish and allowed to cool at room temperature. Then, 2 g of fresh stool are placed in the center of the agar plate and incubated in an incubator (26–33 °C). The plates are examined for characteristic tracks of larvae movement every 24 h, for up to 72 h. Place the agar plate under a light microscope and examine for the presence of motile larvae which determines a positive test [36,37].

### 4.5. Baermann Technique

For this technique, a fecal sample is suspended in a bowl filled with warm water for up to 2 h. This allows the larvae to migrate from the feces into the surrounding water environment. It requires about 10 g of stool placed in the center of a double layered cheesecloth. This is then suspended on a piece of wire gauze and two layers of cotton gauze in a 6 inch plastic glass funnel that is attached with rubber tubing and a pinch of clamp attached at the bottom. The glass funnel is filled with warm water and the preparation is left to stand for 2 h. After 2 h of incubation, the clamp is opened to collect 10 mL of the liquid and centrifuge the tube. After centrifugation, a drop of the sediment is transferred onto a glass slide and mixed with one drop of Lugol’s iodine solution. The added iodine helps to make the visualization of the larvae easier under the microscope using the low-power objective (x10) [35,38].

### 4.6. Water Emergence Technique for Detecting Strongyloides Larvae in Feces

This method requires the use of a fresh stool sample. A deep hole is made in the center of the stool specimen and filled with warm water. The incubation period at 35–37 °C in an incubator for up to 3 h permits the larvae to migrate out of the feces into the surrounding warm water. Some of the water is pipetted and transferred onto a glass slide and a cover glass is placed over it to make a thin preparation. An alternative approach involves collecting the water completely which is then transferred into a conical tube. The tube is centrifuged at 1500 rpm for 2 min and the sediment transferred onto a glass slide. The preparation is microscopically examined for motile larvae using the 10x objective. This is a cost-effective method suitable to be used in resource-limited settings [23].

### 4.7. Harada-Mori Technique

**Figure 5:**
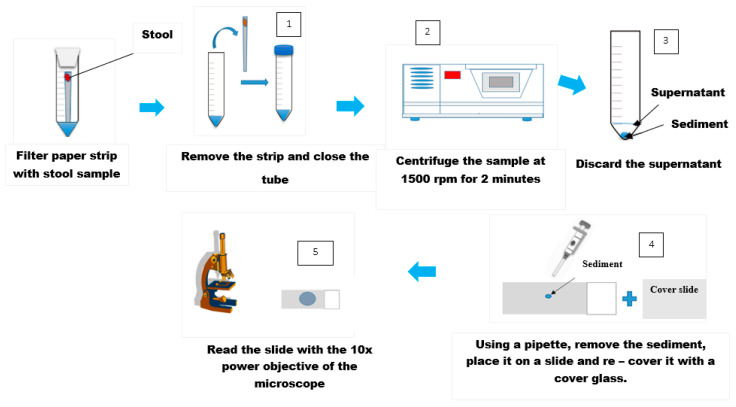
Operating steps of the Harada-Mori technique. Adapted from open source web images.

This technique was initially introduced in 1955 [39]. It involves the use of a filter paper to which the feces are added and placed in a test tube. Several modifications of the technique have been described [40,41]. Continuously adding water soaks the filter paper and thus provides moisturized conditions that are favorable for the ova to hatch and the development of the larvae to occur. This technique is very simple and more efficient when fresh fecal samples are used. It involves cutting a filter paper into narrow strips of about 5 inches with slightly tapered ends. One gram of feces is transferred onto the center of the strip. After adding up to 4 mL of distilled water into a 15 mL centrifuge tube, the filter paper strip containing the stool is inserted into the tube such that the tapered end merely touches the bottom of the tube, while the water level is slightly below the fecal spot. The tube is then screw-capped, cork stoppered of cotton plugged. The tubes are then kept in an upright position at 25 to 28 °C for up to 10 days with daily checks of the water level for evaporation. A smear is then prepared after 10 days incubation by withdrawing the fluid to a glass slide and examining with the 10x objective to detect infective third stage motile larvae of Hookworm or free-living and infective third stage filariform larvae of *S. stercoralis.* Alternative procedures involve a centrifugation step as shown in Figure 5.

### 4.8. Merthiolate-Iodine-Formaldehyde-Concentration Technique (MIFC)

The MIF technique is a concentration-based method that requires a centrifuge. It is suitable to detect protozoan parasites, but its sensitivity is limited for the detection of other helminths. The procedure employs the MIF solution (50 mL formaldehyde at 37%, 10 mL glycerin at 87%, filled to 1 L with distilled water as stock solution I) as a preservative and staining (with 2 g potassium iodide in 10 mL distilled water as stock solution II). Ether is added to dissolve the fecal fats. The preserved fecal specimen is prepared as described by Sapero and Lawless [42]. It involves mixing the MIF preserved specimen by shaking vigorously for five seconds. The mixture is strained through a gauze into a 15 mL centrifuge tube. Up to 3 mL of ether is added to the centrifuge tube and the tube is closed with a rubber stopper. The tube is shaken by vortexing to mix. The ether used should be refrigerated to reduce volatilization. The stopper is removed and let stand for two minutes, then centrifuged for 5 min at 1500 rpm to obtain four layers in the tube (an ether layer, fecal debris, formalin, and sediment). The sediment contains protozoa and helminth eggs. The fecal plug is carefully removed, and the sediment is separated from the rest of the solution on top. A drop of well mixed sediment is transferred and put onto a glass slide and examined under the microscope. The time required to prepare the MIFC specimen for examination is about four minutes [42,43].

### 4.9. Flotation Techniques

Flotation tests are mostly used for the detection of eggs of different parasitic worms that are shed in feces. The principle of the fecal flotation of parasite eggs makes use of the lower specific gravity of the eggs compared to that of the flotation solutions (FS). The various FS vary in specific gravity depending on the formulation and could range from 1.18 to 1.27. Most parasite eggs have a specific gravity (sg) of 1.05–1.20 which allows them to float while large fecal debris which are denser will sink to the bottom.

### 4.10. Zinc Sulfate Flotation Technique

This technique is recommended for concentrating eggs of *Trichuris trichiura* but also cysts of *Giardia lamblia* and *Entamoeba histolytica*. It is less time consuming when compared to the other flotation techniques. The method involves the use of a zinc sulfate solution (sg: 1.180–1.200). One gram of feces is emulsified in tap water and strained to remove the fecal debris. The filtrate is then centrifuged, and the sediment is suspended in 4 mL of ZnSO4 solution. The suspension is allowed to stand for 30–45 min for the eggs and cyst to float to the top. A cover glass is placed on top of the tube to collect the eggs/larvae, which are transferred onto a glass slide to be examined under a microscope [24,44].

### 4.11. Saturated Sodium Chloride Flotation Technique

In field surveys, this technique represents a useful and inexpensive tool most used for concentrating the eggs of Hookworm and *A. lumbricoides*. It has the same principle as the zinc sulfate technique described above with the only difference in the choice of the FS. The FS used in this technique is a saturated sodium chloride solution [24].

### 4.12. FLOTAC Techniques for Detecting Helminths Eggs

Recent studies suggest the use of the FLOTAC technique for the diagnosis of STHs in humans. These techniques have been used extensively in veterinary fields [45,46,47]. It is an innovative method to count fecal eggs by combining flotation, centrifugation and translation using a single FLOTAC apparatus. There exist different FLOTAC protocols depending on the FS used. These include the basic, dual, double and pellet techniques that require up to 1 g of stool leading to an improved theoretical analytic sensitivity of two eggs per gram (EPG). The amount of stool used is about 24-fold higher than for the Kato-Katz technique (41.7 mg), an important factor that explains the higher sensitivity of the FLOTAC technique [30]. Moreover, there is the possibility to use stool samples preserved up to 83 days [48]. The technique is less time consuming compared to the Kato-Katz technique requiring just about 12–15 min from preparation to microscopy analysis. It involves accurately weighing up to 1 g or more of the fecal sample taken from a large amount of fecal material and thoroughly homogenizing it in tap water. A wire mesh is used to filter through the homogenized suspension into a conic tube and the tube is centrifuged for 3 min at 1500 rpm. After centrifugation, the supernatant is discarded and the tube refilled with the FS of choice, and finally homogenized to obtain a suspension and the suspension is used to fill the two flotation chambers of the FLOTAC apparatus. The FLOTAC apparatus is closed and centrifuged again for 5 min at 1000 rpm. After centrifugation and the translation of the top parts of the flotation chambers, they can be read under the microscope.

### 4.13. Stoll’s Dilution Egg-Counting Technique

Unlike other methods, Stoll’s technique has the advantage of being rapid, inexpensive and offers the possibility of egg quantification. In this technique, 3 g of feces is weighed and a one in 15 dilutions with water in a screw-cap container is made. The use of sodium hydroxide 0.1 mol/L in place of water is recommended when using formed stool. The container should be capped and well shaken to homogenize. Using a Pasteur pipette up to 0.15 mL of the suspension is transferred onto a glass slide, covered with a cover slip, and examined systematically under a microscope. The final quantification is by multiplying the egg count by 100 to obtain the number of eggs per gram of feces [24].

### 4.14. McMaster Method for Quantitative Fecal Examination

**Figure 6:**
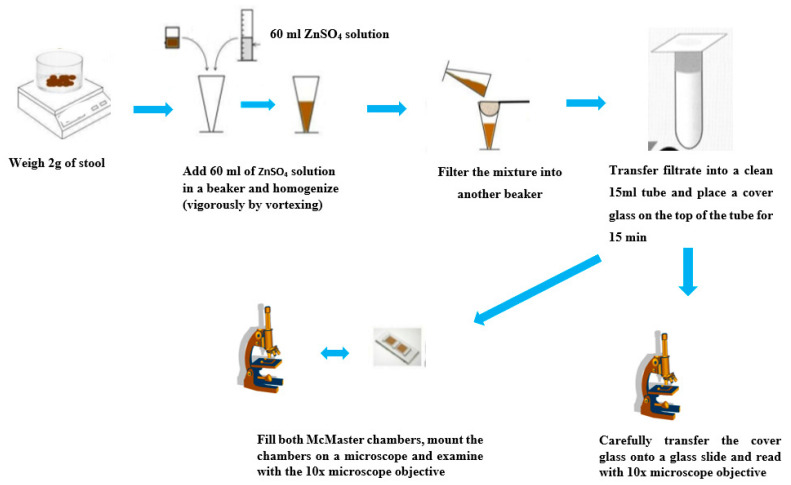
Operating steps of the McMaster technique. Adapted from open source web images.

This technique provides a quantitative determination of the burden of nematode worm infections expressed in eggs per gram of feces. It is comparatively fast and floating eggs can be easily recovered free of debris and loaded into a counting chamber. The procedure involves weighing up to 2 g of feces and transferring into a beaker containing 60 mL of ZnSO4 FS (sg: 1.18–1.20). An alternative is to weigh 2 g of feces into 30 mL of saturated salt solution (sg: 1.2). After stirring vigorously to homogenize the feces, they are then sieved through a cheesecloth or wire-mesh into a second container. The filtrate is transferred into a clean 15 mL tube, a cover slip is placed on top and then is allowed to stand for 15 min. Following this, the cover slip is carefully transferred onto a glass slide and read under the 10x power objective of the microscope. The suspension is re-homogenized and both chambers of the McMaster slide are filled using a pipette. The chambers are allowed to stand for up to 3 min to allow the eggs to float to the top, while the debris fall to the bottom of the chamber and are examined using the 10x power objective of the microscope. Only the eggs that fall within the gridded area of both sides of the chamber are counted. The final quantification is by multiplying the egg count by 100 to obtain the number of eggs per gram of feces [25]. Several studies have been performed to evaluate the sensitivity of this technique. When compared to the Kato-Katz technique for the detection of soil-transmitted helminths, the McMaster was found to be more sensitive and provided accurate efficacy results [49,50]. Figure 6 shows the operating steps of the McMaster method.

### 4.15. Antigen Detection

The methods described so far are based on the detection of parasitic elements (eggs/cysts/larvae) in stool. However, some studies have described the use of coproantigens captured in an ELISA assay. The underlying principle in the use of these assays relies on the capture of parasites excretory/secretory (E/S) proteins using rabbit anti-E/S polyclonal antibodies [51,52]. These methods have been described to be effective in the diagnosis of *S. stercoralis* and hookworm infections. However, the methods based on antigen detection have not been widely used in STH diagnosis as they have been with other parasites such as *Plasmodium species* and other protozoan parasites.

### 4.16. Polymerase Chain Reaction (PCR) Technique

Microscopy techniques require personnel expertise, multiple stool sampling and species-specific concentration and staining methods to improve performance. The limitations of these techniques with regards to specificity, sensitivity, intra-specimen variability of egg counts, low-infection intensities and other factors mentioned earlier have led to an increased use of PCR assays for intestinal parasites diagnosis [53,54]. Nucleic acid-based detection has had tremendous success in virology and bacteriology and now increasing efforts are geared towards their use as first-line diagnostic tools for clinical parasitology. There are however growing concerns that these techniques might replace microscopy and could have (possible) clinical drawbacks and the beauty of microscopy that allows visualizing the different forms of parasitic elements might be lost [55]. Different protocols have been developed for PCR assays based on single, nested, real-time qPCR, and multiplex PCR [56,57,58]. Depending on the target interest and available resources the choice of the PCR can vary from a simple semi-quantitative to quantitative real-time PCR. The steps in a PCR reaction involves a repetitive cycle of DNA denaturation, primer binding and extension by a Thermo resistant Taq DNA polymerase [59]. PCR methods have higher sensitivities and specificities, requiring very small amounts starting DNA material [60]. A major caveat is to know the DNA sequence of the target to design primers for amplification. Other drawbacks include DNA damage in stool samples, the amplification of contaminants [61], well trained personnel and the lack of infrastructure in low-resource settings. However, with more and more technicians trained in molecular diagnostics and with recent advances in technology that include automation at various stages of the PCR process, issues related to contamination have been greatly reduced as these techniques are being optimized.

Loop-mediated isothermal amplification (LAMP) are novel techniques capable of amplifying DNA with higher specificity and efficiency. The LAMP technique allows DNA amplification in a one step process under isothermal conditions. Amplification takes place at a constant temperature of 60–65 °C and requires the use of two, three or four sets of primers and a strand-displacement DNA polymerase with replication activity. These conditions lead to the accumulation of large quantities of target DNA as compared to PCR-based amplification, thus increasing the specificity and sensitivity of the LAMP technique [62,63]. Following its first reported use in 2000, the LAMP technique has been used extensively across various fields for the detection of bacterial, viral, fungal and parasitic infections. They have a potential application as screening assays or as point of care diagnostic platforms. A colorimetric isothermal assay was recently developed using SmartAmp2 to identify hookworm (*N. americanus*)*, T. trichuria* and *A. lumbricoides* with an observed high sensitivity [64]. The LAMP technique can have wide application in low- and middle-income countries (LMIC) where the goal is to reduce morbidity due to STH infections.

The digital PCR method is different from the conventional PCR in that the PCR reactions are performed in tens of thousands of nano-liter sized droplets each leading to a separate PCR reaction. This partitioning greatly improves the precision of the technique and thus increases the efficiency of the quantification of the target DNA [65]. Both qPCR and digital PCR were shown to be able to detect very low amounts of *A. lumbricoides* with high sensitivities from reclaimed water in a wastewater depuration test [66]. The application of these techniques to detect other STH infections especially in low-intensity infection settings will be very useful to control transmission.

The multiplex qPCR method allows the quantification and detection of several target DNA sequences simultaneously. DNA amplification occurs in real time using a combination of multiple primer sets. In the past decade several multiplex qPCR assays have been developed for the detection of STH infections. Using species-specific primers/probes, studies have shown an increased sensitivity of detecting up to eight gastrointestinal parasite pathogens [67]. Given that global efforts are in favor of committing resources towards the control and elimination of STH in LMIC, there is an urgent need to compliment these efforts with alternative diagnostic assays that can demonstrate excellent run-to-run consistency, reproducibility and are high-throughput [68] or they have a high sensitivity and specificity, but are also potentially cheap and can be used in limited-resource settings [69].

Table 1 provides a summary of the diagnostic values of the above described methods. It also highlights which techniques are suitable for the detection of specific STH parasites. Meanwhile, Table 2 describes the advantages and disadvantages of each technique.

## 5. How to Choose a Diagnostic Technique

It is hard to imagine that despite the considerable progress over several decades to control the spread of parasitic diseases in the developing countries following a partnership between different governments and international organizations in such as the WHO, it is still not possible to have an adequate and accurate diagnostic test. Most of the data describing the distribution, prevalence and the burden of parasite infections in endemic communities were obtained using the methods described above. Their performance characteristics (sensitivities and specificities) thus determine the accuracy of such data [70]. In low-intensity infections, most of these tests do not perform well in population assessment, especially after multiple rounds of MDA have been used, reducing infection levels in endemic communities. The consensus is to harmonize diagnostic protocols to improved STH diagnosis. This means the development of more new sensitive techniques or the optimization of available techniques. If one has to choose from the available diagnostic assays, several aspects are to be considered including; the objective of the diagnostic test, the accuracy of the diagnostic tool (sensitivity and specificity) and a balance between the quality and the cost-effectiveness (precision, simplicity, and robustness) as well as the time and effort. Some of these techniques are quantitative and provide an advantage when used to measure morbidity. They can thus be very useful to assess the reduction of infections in control programs such as the WHO goal to reduce infections to less than 1%.

The issues we outline here are intended to raise awareness of the need to optimize helminth diagnostic techniques, to make a balance between the operational cost-effectiveness of the techniques that will suit the nature of control that is intended to be achieved.

### 5.1. Operational Cost and Infrastructure

The burden of infection is higher in endemic areas with limited resources. To this effect, the set goal is usually aimed at targeting morbidity control through mass treatment. The choice of a diagnostic technique is almost always based on cost and simplicity with limited emphasis on the sensitivity of the technique [70]. The MIFC, agar plate, Baermann, formol-ether, McMaster, FLOTAC and PCR techniques which require expensive materials and more sophisticated equipment are therefore not particularly suitable for field surveys, or the rapid identification of those most in need of treatment. Recent advances in molecular diagnostics including the use of rapid diagnostic tests (RDT) and smart optical devices to detect other parasites such as malaria parasites have shown tremendous progress in improving diagnosis at the point of care [71,72,73]. Expanding these techniques to detect helminth parasites such as hookworms will be beneficial mostly in endemic areas where the focus is to reduce transmission.

### 5.2. Sensitivity and Specificity

The need for a precise diagnostic test is the most important aspect when transmission control is set as the target goal. Several of the aforementioned techniques demonstrate a low sensitivity since they often fail to detect infections in low-infection intensity settings [74]. Flotation techniques are less sensitive because not all nematodes can be well concentrated. When stool samples contain much fecal fat, these techniques become difficult to realize [28]. The sensitivities of the direct smear examination and the Kato-Katz techniques are reduced when a single stool sample is examined. As such, most protocols suggest the use of multiple stool samples in low-intensity infection settings [19]. Sensitivity increases when the analysis is repeated with several samples from 52% (single day) to 79% (consecutive day) [75]. Usually, very small amounts of feces are examined using the Kato-Katz technique (41.7 mg). This small amount consequently underlies the techniques’ low analytic sensitivity [30]. Other factors that affect the sensitivity of the Kato-Katz technique are day-to-day changes in the egg excretion [31,32], time delays from when the feces was produced, collected, and processed in the laboratories, and the rapid clearing of hookworm eggs [33,34]. The MIFC is more suitable for intestinal protozoa but lacks sensitivity in detecting or quantifying helminth eggs, especially hookworm eggs [30]. The larvae of hookworm and *S. stercoralis* are sometimes missed in the usual Kato-Katz and FLOTAC techniques thus the Agar plate, Baermann, and Harada-Mori techniques remain the best choice for detecting these parasites. The FLOTAC techniques were found to be more sensitive when compared to multiple Kato-Katz thick smears for the diagnosis of hookworms, *A. lumbricoides* and *T. trichiura* infections [46,48]. The FLOTAC technique therefore could help solving challenges posed by currently available techniques, but also presents some limitations ranging from cost to sensitivity to detect the larvae of *S. stercoralis.* Moreover, as for all other fecal concentration methods requiring the flotation of parasitic elements, the FS used can greatly influence the sensitivity of the FLOTAC technique [30]. Although rapid diagnostic tests (RDTs) have shown tremendous success in detecting the antigens of some protozoan parasites, the development of such assays to detect antigens of intestinal helminths could still be problematic when one considers issues concerning the cross-reaction that can affect the sensitivity of such assays. The use of bio-informatic tools to identify biomarkers of STH parasites should be encouraged. Such biomarkers can be developed into antigen/antibody tests that could be used as a tool for STH surveillance to characterize different endemic populations and to effectively measure or monitor the progress of control programs and for vaccine development.

### 5.3. Safety Issues and Personnel Qualification

When aiming at morbidity control, further diagnostic challenges concerning stool examination should be considered. As mentioned earlier, infections with multiple species of parasites are usually common and as explained earlier no single technique can provide an accurate diagnosis of all different parasite species. Therefore, the performances of these techniques will greatly improve when laboratory personnel are well trained and adequate quality-control measures are put in place in limited-resource settings [28]. There are also concerns related to safety, time delays between specimen collection, transportation to the laboratory and analysis, laboratory infrastructure and the labor-intensity required for the performance of the technique. The zinc sulphate technique and saturated NaCl are less safe techniques since the reagents used do not inactivate fecal pathogens and thus represent a potential source of contamination for laboratory personnel. The ether used in the formol-ether technique is highly flammable. It is recommended to refrigerate the ether before use to reduce volatility or to use a less flammable ethyl-acetate, meanwhile, the Kato-Katz is unhygienic [38]. The agar plate and Baermann techniques are more labor intensive, time consuming and show similar sensitivities [50]. The Baermann technique is cumbersome whereas the agar plate requires the more skillful and careful manipulation of the samples to prevent the percutaneous infection of the laboratory personnel [38,50]. The performance of the FLOTAC technique requires a high level of competence from laboratory personnel. The use of point-of-care (POC) immunoassay platforms such as those used for Plasmodium species and Schistosome detection may be a better alternative. Such POCs can be designed to accommodate the multiplexing capacity for the detection of pan-STH species. Laboratory-based nucleic acid amplification tests (NAAT) are a valuable alternative. However, the full evaluation and harmonization of PCR protocols is recommended to boost the performance of these techniques.

## 6. The Way Forward

The STH infections in areas of high endemicity have huge socio-economic and developmental consequences for infected populations. Despite global strategies implemented through the WHO and various NGO partnerships to reduce the burden of infections through mass drug administrations and morbidity control, a more effective approach would be to integrate laboratory testing using highly sensitive and specific techniques as a useful adjunct to clinical examinations and sound imaging techniques. We hereby have undertaken a review of some of the currently available intestinal helminth diagnostic techniques and their limitations. Stool examination by microscopy techniques provides an acceptable measure to assess infection levels in highly endemic areas. However, its relevance in areas of low endemicity is limited. Therefore, other more sensitive techniques such as the PCR are required in such areas. On the other hand, with proper training of the laboratory personnel, the sensitivity of microscopy-based techniques can be greatly improved. Molecular diagnosis with PCR or antigen (Ag) detection, although initially deemed as a superior/more adequate assay for diagnosis, some studies had suggested that it is only marginally more sensitive than microscopic stool examination [28]. As an alternative, one would recommend the FLOTAC techniques which have shown higher sensitivities than the currently available methods. Taken together, the PCR is currently the most accepted technique because of its slightly higher sensitivity and specificity, but with the non-negligible fact of its high cost and limited availability for resource-limited settings.

If we want the ongoing control strategies to succeed, it will be imperative to develop and implement clear-cut protocols, to identify good biomarkers that can be multiplexed to detect all STHs and to perform more the rigorous validation of new diagnostic assays through multi-country (site) studies especially in low endemic settings.

## Figures and Tables

**Table 1:** Fecal concentration techniques and their limits of detection of soil-transmitted helminths (STHs).

Parasite	Formol-Ether	Kato-Katz	MIFC	McMaster	FLOTAC	Sat. NaCl	ZnSO_4_	Agar Plate/Baermann
**Eggs**								
*A. lumbricoides*	++	++	-	+	+	+	-	-
*T. trichiura*	++	++	-	+	+	-	+	-
*Hookworm*	++	+	+	+	+	+	-	-
**Larvae**								
*Strongyloides*	+	-	-	-	-	-	-	++
*Hookworm*	+	-	+	-	-	-	-	++

++: good diagnostic value, +: low sensitivity, -: limited or no diagnostic value.

**Table 2:** Advantages and disadvantages of the diagnostic techniques to detect STHs.

Technique	Advantages	Disadvantages
Kato-Katz	Simple to operate and cost-effective Quantitative	Requires small stool amounts, thus low analytic sensitivity Requires fresh samples
Ether-based concentration techniques	Stool samples can be preserved in formol Eggs of helminths and cyst of protozoa can be detected	centrifugation can destroy eggs of hookworms cannot be performed in laboratories with minimal infrastructure Only qualitative
Flotation techniques: (ZnSO_4_ and saturated NaCl)	Simplicity and low cost	Lack of precision owing to the absence of a grid on the cover slides Only qualitative
Harada-Mori	Simple and cost-effective Allows both parasitic and free-living forms to be detected	It requires distinction from parasitic to free-living forms. Not suitable for refrigerated samples Too much time required to obtain results and only suitable in field surveys where rapid results are not that important
McMaster techniques	Relatively fast and simple to perform	Choice of flotation solution may influence results Requires the use of a counting chamber which might not be readily available in resource-limited settings It has a detection limit of 100 eggs per gram (EPG) unless multiple counts are done on the same sample
FLOTAC	Both fresh and preserved samples can be used for analysis. Eggs of helminths and cysts of protozoa can be detected High sensitivity and accuracy	Requires centrifuges designed to hold the FLOTAC apparatus Well trained laboratory personnel are required to perform this technique
Serology (antibody detection)	Used to demonstrate exposure Confirm clinical findings	In endemic areas antibody test generates many false positives due to previous exposure
Antigen detection	Sensitive in detected coproantigens of S. stercoralis	Production of these tests has not been extended to other STHs
PCR	Increased sensitivity and specificity Species and strain level identification of parasites is possible. Molecular epidemiology to monitor transmission patterns	Requires well equipped laboratory infrastructure and well-trained personnel more expensive compared to the Kato-Katz technique Contamination can lead to false positive
